# Impacts of Reducing Protein Content in Milk Replacer on Growth Performance and Health of Young Calves

**DOI:** 10.3390/ani12141756

**Published:** 2022-07-08

**Authors:** Dana Carina Schubert, Bussarakam Chuppava, Sandra Hoffmans, Martin Pries, Christian Visscher, Josef Kamphues, Amr Abd El-Wahab

**Affiliations:** 1 Institute for Animal Nutrition, University of Veterinary Medicine Hannover, Foundation, Bischofsholer Damm 15, D-30173 Hannover, Germany; bussarakam.chuppava@tiho-hannover.de (B.C.); sekretariat-tierernaehrung@tiho-hannover.de (S.H.); christian.visscher@tiho-hannover.de (C.V.); josef.kamphues@tiho-hannover.de (J.K.); amrwahab5@mans.edu.eg (A.A.E.-W.); 2 Landwirtschaftskammer Nordrhein-Westfalen, Versuchs- und Bildungszentrum Landwirtschaft, Haus Riswick, D-47533 Kleve, Germany; martin.pries@lwk.nrw.de; 3 Department of Nutrition and Nutritional Deficiency Diseases, Faculty of Veterinary Medicine, Mansoura University, Mansoura 35516, Egypt

**Keywords:** calf nutrition, milk replacer, protein reduction, health

## Abstract

**Simple Summary:**

The appropriate protein percentages in calf milk replacer (MR) is a key factor in determining the optimal growth of young calves. We hypothesized that elevated MR feeding with a lower protein content may not negatively affect the performance, metabolic traits, and health of the calves. The question on is: how much protein is required by calves in order to achieve an intended growth rate? In the present study, the average daily gain was similar between both groups fed MR of 19% and 22% protein, although crude protein intake was reduced in the group fed MR of 19% protein. Even though the mean dry matter content in the feces of group fed MR of 19% protein was partly lower, the incidence of diarrhea was not greater in this group. Both groups fed MR of 19% and 22% protein had very great serum insulin-like growth factor 1 (IGF-1) concentrations. This confirms that both groups, i.e., also the animals fed with a crude protein-reduced MR, were adequately supplied with nutrients. Furthermore, the lower serum urea concentration in the 22% protein in MR calves indicates that they probably had a greater body protein accretion and the proportion of amino acids for energy supply was lower than in the 19% protein in MR calves. Thus, the urea content in the blood serum indicated that a higher protein intake during the pre-transition period is feasible with a higher protein content in the MR.

**Abstract:**

In the present study, a drinking amount of 10 L of milk replacer (MR) was allowed to dairy calves in order to approach the natural drinking behavior. The question is: how much protein is required by calves in order to achieve an intended growth rate? For this reason, sixty-eight pre-weaned Holstein calves were divided into two groups and fed with 10 L/d of MR containing either 22% protein (MR22) or 19% protein (MR19) at an almost comparable energy intake. Effects on performance, metabolic status, and health were compared. Feed intake, growth performance, and health status were monitored during the pre-transition, transition, and postweaning phase (until 157 d of age). Total feed intake, and intake of MR, body weight (BW), and average daily gain (ADG) were not significantly different between MR22 and MR19 during the entire experimental period (*p* > 0.05). At d 42, calves in MR19 group showed greater serum levels of growth hormone (16.2 vs. 22.2 ng/mL; *p* = 0.02), insulin-like growth factor 1 (262 vs. 291 ng/mL; *p* = 0.03), and urea (2.86 vs. 3.04 mmol/L; *p* < 0.01). The results of the present study suggested that when high amounts of MR are provided, the protein content in MR can be reduced to 19% without any adverse effects on growth performance as well as on health status of dairy calves.

## 1. Introduction

Well balanced nutrition (energy and nutrient supply) is necessary for optimum performance, health, and welfare of calves [1]. Calf health, welfare, and performance in the first few months of life are important for reducing calf mortality and for increasing the lifetime productivity of dairy cows [2,3]. Several studies have focused on the milk replacer (MR) feeding regimen in newborn calves to stimulate postnatal growth and development through intensive nutrient intake to enhance health, organ development, structural growth, and well-being [4,5,6]. Generally, feeding practices provided daily milk allowances of approximately 10% of calf body weight (BW), primarily to increase solid-feed intake to facilitate rumen development for earlier weaning [7]. These restricted feeding practices may affect the welfare issues and the growth potential of calves [7,8]. A calf left with its dam will suckle on average 7 to 10 times a day and consume much more milk in comparison to those fed a total of about 10% of the calf’s BW [9]. During the pre-weaning period, calves fed milk or MR at a high rate will have an increased rate of weight gain [4,10,11,12]. Jasper and Weary [11] found that ad libitum fed calves gained 63% more weight than the conventionally fed calves before weaning, resulting in a 10.5 kg weight advantage on d 35.

In comparison to whole milk containing about 24% crude protein (CP), typical commercial MR formulations contain 20 to 28% CP [4,13,14]. In addition to offering higher amounts, it was advised that the MRs have a higher protein content (26% to 28% CP) to meet the protein needs for rapid muscular growth [10]. Diaz et al. [10] demonstrated that feeding a 30% protein MR increased lean tissue deposition without fattening. Moreover, protein is needed for several body functions at this and every age (bone formation, immune system etc.). The goal of this “accelerated” development regimen is to take advantage of young calves’ high lean tissue growth potential and encourage more lean growth without fattening them. Calves fed high amounts of MR (>900 g/d) with an increased protein content up to 26% and a low fat content (16%) resulted in a high growth rate with low body fat content [15]. Nonetheless, the high cost of the ingredients, especially milk protein, makes the use of low protein content an increasing issue in the formulation of MR. Accordingly, the current economic climate presents a challenge to dairy producers to rear calves of high profitability and welfare outcomes while minimizing the input costs of the enterprise [15]. The question remains which protein content in MR feeding is necessary to maximize calf growth and/or health.

Starting with the preweaning period of calves, the feeding strategy affects the maturation of the postnatal somatotropic axis [16,17]. In cattle, the insulin-like growth factor (IGF) axis plays a crucial role in postnatal development and growth [18,19,20,21]. Increased protein and energy intake from increased milk or MR feeding enhances IGF-I secretion and somatotropic axis maturation, as the somatotropic axis is dependent on nutritional intake [22,23,24]. Thus, calves might benefit via improved growth from a stimulated somatotropic axis as a result of intensive MR feeding.

Therefore, there is great interest in understanding the consequences of MR feeding regimes on the pre-weaning growth and health of calves. We hypothesized that elevated MR feeding with a lower protein content may not negatively affect the performance, metabolic traits, and health of the calves. The question is: how much protein is required by a calf in order to achieve an intended growth rate. For this reason, sixty-eight newborn Holstein calves were divided into two groups and fed with 10 L/d of MR containing either 22% protein (MR22) or 19% protein (MR19) at an almost comparable energy intake. Effects on performance, metabolic status, and health were compared.

## 2. Materials and Methods

The experiments were conducted in accordance with German regulations and approved by the North Rhine-Westphalia State Agency for Nature, Environment, and Consumer Protection in Germany (reference: AZ 84-02.05.20.13.024).

### 2.1. Animals, Housing, and Zootechnical Measures

The experimental study was conducted using 68 calves (34 males and 34 females) of the German Holstein breed from the Agricultural Research and Teaching Centre Haus Riswick of the Chamber of Agriculture in North Rhine-Westphalia, Germany.

After birth, the calves were separated from their mothers and moved to individual boxes (0.95 m × 1.25 m) littered with straw for their first week of life. At d 8 of life, the calves were divided into two different groups (MR22: *n* = 35; MR19: *n* = 33), and moved into group housing to groups of 16-18 calves. The groups were equally divided to four different pens. Two of these pens were identical in construction. Pen A and Pen B consisted of a 9 m × 3.4 m straw littered area, and a 9 m × 1.5 m walkway with slatted concrete adjacent to the feed table. In Pen C and Pen D, the littered straw area measured 9 m × 6.2 m and the stand area between straw area and feed table was 9 m × 1.3 m and had a solid concrete floor. The two pens of each group were continuously filled up so that each group pen was completed in as short a time frame as possible. For group MR22 it took 10 and 18 days, respectively, and for group MR19 it took 12 and 17 days, respectively. The calves remained within the groups until d 157 of life.

The zootechnical measures after birth included spraying the navel with oxytetracyclin hydrochloride spray (Engemycin^TM^ Spray 3.84%, MSD Tiergesundheit, Unterschleißheim, Germany), and offering colostrum. At the first day of life, ear tags were applied and 5 mL of iron (III) hydroxide-dextran complex (100 mg/mL, Belfer^®^, bela-pharm GmbH & Co. KG, Vechta, Germany), 5 mL vitamin E/selenium supplement (Vitamin-E-Selen ad us. vet., 150 + 1.1 mg/mL, aniMedica GmbH, a LIVISTO company, Senden, Germany), and 5 mL vitamin B complex (Vitamin-B-Komplex pro inj., Serumwerk Bernburg AG, Bernburg, Germany; list of vitamin concentration in Table S1) were injected subcutaneously. Dehorning of calves that were not genetically hornless was performed in the 3rd to 5th wk of life. At the end of the trial, the females were used to replace old cows from the herd.

### 2.2. Diets, Feeding Concept, and Experimental Design

Directly after birth, calves were offered colostrum. Six and 12 h later, calves were offered two times 2 L of colostrum with a teat bottle. The quality of MR was not recorded. Thereafter, until d 7 of life, calves were fed ad libitum with acidified colostrum or whole milk by means of teat buckets. Refusals were recorded volumetrically. When the calves were housed in groups, calves were individually recognized by means of transponders at automatic milk-feeders and received (up to) 10 L of MR that contained either 22% (control group: MR22) or 19% protein (experimental group: MR19). Calves were offered up to 349 and 296 g CP by MR for control and experimental groups, respectively. The components and chemical composition of the MR are presented in Table 1. The chemical composition as well as type and amount of feed additives are presented in the Supplementary Materials Table S2.

For all groups, the MR was mixed at a concentration of 160 g/L until d 42. In the following weeks, the concentration of MR was reduced to 125 g/L and the amount of MR was linearly reduced by 2.5 L per week over four weeks. Within a week, a gradual reduction in 0.5 L increments occurred on five of seven days. On two days in each week (day 3 and day 6), the quantity provided did not change. The corresponding milk quantities could be both retrieved and registered at the automatic feeders (SA 2000, Förster-Technik GmbH, Engen, Germany) using transponders on the calves’ neck.

In addition to MR, the groups received a mixed calf feed (MCF) and total mixed ration (TMR) of the dairy cows. The MCF consisted of 87% of a calf concentrate and 13% of chopped barley straw. The TMR was designed for dairy cows to meet the energy and nutrient requirements for 26 kg energy corrected milk (ECM) and was composed of corn silage (37% of DM), grass silage (26% of DM), mixed concentrates (16% of DM), rapeseed extraction meal (11% of DM), straw (7% of DM) and mineral feed (2% of DM). The chemical composition of the MCF and TMR is displayed in Table 2. The MCF was only offered ad libitum until the youngest calf in each pen was 77 days-old of life. From d 78–97 of life of the youngest calf in each pen, MCF was restricted to 2 kg/animal/d and from d 98-117 of life to 1 kg/animal/d. From d 78, calves received TMR ad libitum. The change in the feeding regime was based on the age of the youngest animal in the group.

### 2.3. Measurement and Sampling Procedure

In both groups, the daily intake of MR was recorded for each animal individually, whereas the intake of solid feeds (MCF and/or TMR) was recorded on a group basis. Body weight (BW), sacral height (SH), and rectal temperature (RT) were measured directly after birth. In the following, the parameters BW, RT, and SH as well as fecal samples were collected from the calves once a week for the first ten weeks of the trial (until age 77 d). Two further samplings took place at 117 and 157 days of age. Blood samples were collected at age 8 d, 42 d, 77 d, and 157 d. However, at the end of the trial (157 d), blood samples were only obtained from half of the calves. Sampling and measurements took place between 08:30 and 11:00 h at the corresponding days. To assess animal health, rectal body temperature was measured daily for the first 21 days and visual inspection of the animals was performed daily throughout the experiment. Suspicious animals were monitored closely and treated with medication if necessary.

The calves were weighed on a cattle scale (weighing range 0-300 kg, resolution = 0.1 kg). A digital thermometer (Vet-Temp, Microlife AG Swiss Corporation, Widnau, Switzerland) was used to measure RT (°C). The average daily gain (ADG) was calculated as gain of weight divided by the number of days. The SH (cm) was recorded with a measuring pole placed vertically next to the calf’s hip. The fecal samples were taken manually from the rectum of the calves and were assessed visually. From the fecal samples, the color (whitish-gray; brownish-green; yellowish-brown; brownish-orange; reddish; dark brown-black) and the consistency (1 = firm; 2 = thick pulpy; 3 = medium pulpy (pasty); 4 = mushy; 5 = loose; 6 = watery) using the score modified after Larson et al. [25] and Chapman et al. [26] were recorded.

The collected fecal samples were frozen at −20 °C until further analysis. Blood was collected by puncturing the jugular vein with a Strauss cannula (1.5 × 43 mm, Dispomed Witt oHG, Gelnhausen, Germany) and monovettes with clot activator for serum collection (10 mL, 95 × 16.8 mm, Sarstedt AG & Co., Nümbrecht, Germany). After centrifugation, the serum samples were stored at −20 °C until further analysis.

For chemical analysis of MR, and components of the MCF (straw and calf concentrates), a collective sample was taken with each new delivery. Furthermore, the on-farm feed components of the TMR (grass and maize silage) were analyzed separately. A sample of the silages was taken weekly from the cutting surface of the silo. Moreover, a pooled sample of the concentrates in the TMR was taken from three deliveries. Dry matter content of the TMR was determined daily at the Agricultural Research and Teaching Center Haus Riswick.

### 2.4. Analytical Methods

The feed materials were analyzed by an accredited service laboratory (LUFA NRW, Germany) in accordance with the official methods of VDLUFA [27]. The energy content of the MR was calculated based on contents of digestible crude protein (dCP), digestible ether extract (dEE), and digestible N-free extract (dNfE) by using the following equation [28]: ME (MJ/kg) = (dCP × 18.1 + dEE × 32.4 + dNfE × 15.2)/1000. The digestibility coefficients were assumed to be 0.98 for CP, 0.97 for crude fat (CF), and 0.96 for NfE [29].

The DM content was determined by drying the samples at 103 °C until weight constancy. In order to determine the pH, 1.0 g feces were mixed with distilled water in the ratio of 1:5. The pH value was then measured in the mixture by means of a previously calibrated pH meter (pH 526 Multical, WTW, a xylem brand, Weilheim, Germany). Sodium content was analyzed in 60 samples (30 samples from MR19 and 30 samples from MR22), which were selected depending on DM content (10 samples highest, 10 samples medium, 10 samples lowest DM content for each group MR19 and MR22, respectively). In this way, the whole range of DM contents that occurred could be investigated with regard to the associated sodium content. The sodium content was analyzed after wet ashing by means of atomic absorption spectrometry (Unicam Solaar 116, Thermo Fisher Scientific GmbH, Schwerte, Germany) according to Schuhknecht and Schinkel [30].

The serum samples were analyzed in the Endocrinological and Clinical Laboratory of the Clinic for Bovine Animals of the University of Veterinary Medicine Hannover, Foundation. The IGF-1 concentration was detected using an enzyme-linked immunosorbent assay (ELISA, DSL-5600, Diagnostic Systems Laboratories, Inc., Webster, TX, USA) after separation from its binding proteins. An ELISA modified from Roh et al. [31] and Kawashima, et al. [32] was used to measure the GH concentration. Insulin levels were measured by radioimmunoassay (DSL-1600, Diagnostic Systems Laboratories, Inc., Webster, TX, USA). Concentrations of glucose was determined in the serum using test strips (OneTouch Vita^®^, Life Scan, Inc., Milpitas, CA, USA). Urea concentrations was determined using a clinic chemical analyzer (ABX Pentra 400, HORIBA Medical, Montpellier, France).

### 2.5. Statistical Analysis

All data were checked for normality using the UNIVARIATE procedure of SAS (version 7.4, SAS Institute Inc., Cary, NC, USA) before any statistical analyses were conducted. As all data followed normal distribution, no data transformation had to be conducted. Statistical analyses were performed by ANOVA using the PROC MIXED procedure of SAS. BW, ADG, SH, fecal DM, fecal pH, and blood metabolites were analyzed as repeated measures. All statistical models were conducted with treatment (MR19, and MR22), age, and a possible interaction between treatment and age as fixed effects. For all the response variables as well as DM content and sodium content depending on fecal DM classification, the means were obtained, and the effect of treatments was performed by F-test in the ANOVA. Significance was declared when *p* < 0.05. The means of rectal temperature were determined by the MEANS procedure as well as the frequencies of the fecal characteristics, e.g., color and consistency were summarized by the FREQ procedure of SAS and assessed using the chi-square test. By using the correlation coefficient, the degree of statistical correlation between fecal sodium content and fecal DM content was assessed. Here, “0” stands for no correlation and “1” or “−1” for a complete correlation. In accordance with Akoglu [33], an absolute value of 0.00–0.29 was considered as poor correlation, 0.3–0.59 as fair correlation, 0.6–0.79 as moderate correlation, 0.8–0.99 as very strong correlation and 1 as perfect correlation. All statements of statistical significance were based upon the level of *p* < 0.05.

## 3. Results

### 3.1. Feed Intake

The mean colostrum intake at the first day of life was 3.5 ± 2.0 L for group MR19 and 3.9 ± 1.7 L for group MR22 and did not statistically differ (*p* = 0.39, Table S3). Generally, it was observed that the MR intake during the pre-transition phase (d 8-42) was not dependent on protein concentration (*p* > 0.05) and amounted to 1310 ± 137 g/d DM in MR22 and in MR19 1300 ± 137 g/d DM, respectively (Table 3). However, with reduced protein content in the MR, the intake of MCF was significantly higher from d 8 to d 42 (Table 3). While, during this period the total CP intake was significantly higher in MR22 compared to MR19 (311 ± 37.8 vs. 276 ± 41.3 g/d; *p* < 0.01), the sum of MR and solid feed intake, defined as total DM intake, did not differ. Whereas, during the transition phase (d 43-77) in both groups, the intake of MR was numerically reduced (MR22 = 555 ± 345; MR19 = 560 ± 353 g/d DM), the MCF intake was numerically increased (MR22 = 1217 ± 664; MR19 = 1344 ± 677 g/d DM). The daily intake of DM was about 4100 g per animal during the postweaning phase (age 78 to 157 d), regardless of the protein concentration in MR previously offered.

### 3.2. Body Weight Gain and Sacral Height

**Figure 1:**
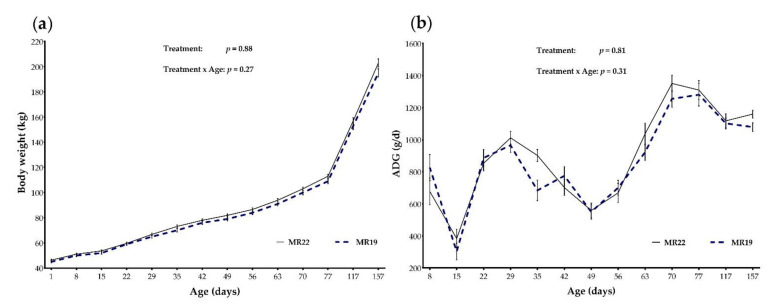
Growth performance represented as means and standard error bars of (**a**) BW (body weight) and (**b**) ADG (average daily gain) of calves fed with milk replacer content 22% CP (MR22; *n* = 35) or 19% CP (MR19; *n* = 33), according to time periods.

BW and ADG (mean ± S.E.) at specific time points of groups MR22 and MR19 are presented in Figure 1. Over the entire experimental period, BW (at birth: MR22 = 46.3 ± 0.78; MR19 = 45.0 ± 0.87; *p* = 0.60 and age d 157: MR22 = 203 ± 3.21; MR19 = 195 ± 3.26 kg; *p* = 0.95) and ADG (age 1–157 d: MR22 = 1003 ± 18.8 g/d, MR19 = 957 ± 19.6 g/d; *p* = 0.93) were not affected by dietary treatment (Figure 1; *p* > 0.05). Mean SH was 82 cm at time of birth and rose to 118 cm at d 157 (Table 4). No differences in dependence on MR protein concentration could be detected for SH during the whole trial (*p* = 0.47).

BW, ADG, and SH was no significant effect of the interaction treatment × age (Figure 1; *p* > 0.05).

### 3.3. Rectal Temperature

On average, body temperatures fluctuated within the physiological range between 38.5 °C and 39.5 °C. Only newborn calves reached average body temperatures slightly below the reference value of 38.5 °C. In the case of body temperatures ≥39.5 °C, a general examination of the calf was carried out and, if necessary, appropriate medication was administered. After a general examination, minor temperature increases could usually be explained by hyperthermia due to high outside temperatures. Of 1147 measurements in group MR22, 113 were above 39.5 °C, which was 9.85% of all measurements. In the MR19 group, 1057 measurements were taken. In 88 measurements, a temperature ≥39.5 °C was detected, which corresponded to 8.33%. The rectal temperature was not significantly different between MR22 and MR19 during the pre-transition phase (*p* = 0.17), transition phase (*p* = 0.09), and postweaning phase (*p* = 0.31).

### 3.4. Fecal Parameters

Fecal parameters include the characteristics of fecal samples (consistency and color) as well as pH, DM, and sodium content of fecal samples.

#### 3.4.1. Characteristics of Fecal Samples

The fecal samples in the pre-transition and transition phase and the postweaning phase appeared most often as greenish-brown and of medium pulpy consistency (Table 5). The groups did not differ in the frequency of observed fecal colors during the entire experimental period. Animals in group MR22 showed a higher frequency of medium pulpy fecal samples than MR19 during the pre-transition and transition phase (MR22: 63.1%; MR19: 57.6%; *p* = 0.02, Table 5) and a higher frequency of thick pulpy consistency during the postweaning phase (MR22: 17.7%; MR19: 5.05%; *p* = 0.02).

#### 3.4.2. DM Content and pH of Fecal Samples

**Figure 2:**
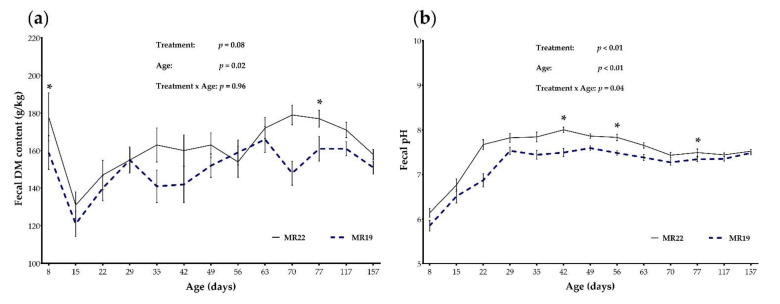
Means and standard error bars of fecal dry matter (DM) content (**a**) and fecal pH (**b**) measured in calves fed with milk replacer content 22% CP (MR22; *n* = 35) or 19% CP (MR19; *n* = 33), according to time periods. * Denotes difference between MR19 and MR22 with *p* < 0.05.

Regarding fecal DM content, there was no significant effect of the interaction treatment × age (*p* = 0.96; Figure 2a), although fecal DM content was affected by age (*p* = 0.02). The fecal DM content was not significantly different between MR22 and MR19 throughout the study, except at d 8 (MR22: 178 g/kg; MR19: 159 g/kg; *p* = 0.03), and d 77 (MR22: 177 g/kg; MR19: 161 g/kg; *p* = 0.05) of life. However, a treatment by age interaction was found for the fecal pH (*p* = 0.04; Figure 2b). The fecal pH did not significantly differ between both groups during entire study except at d 42 (MR22: 8.00; MR19: 7.49; *p* = 0.03), d 56 (MR22: 7.83; MR19: 7.48; *p* = 0.03), and d 77 (MR22: 7.49; MR19: 7.34; *p* = 0.04) of life.

#### 3.4.3. Sodium Content of Fecal Samples

**Figure 3:**
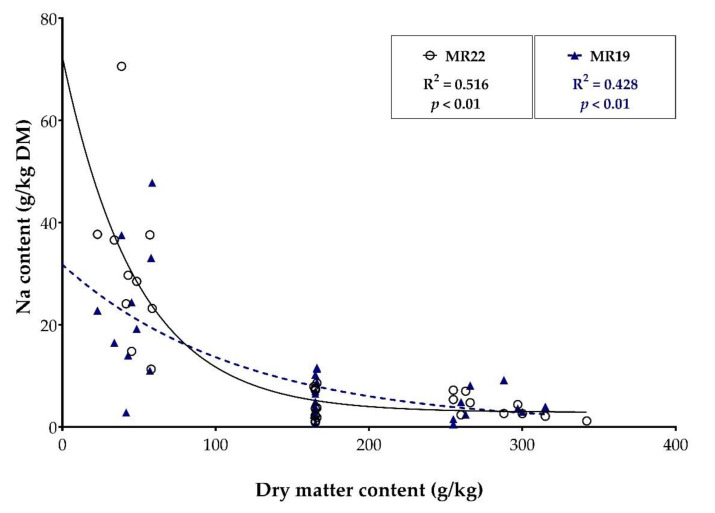
Correlation coefficients with significance levels between the dry matter content (g/kg) and sodium content (g/kg DM) of the fecal samples measured in calves fed with milk replacer content 22% CP (MR22; *n* = 31) or 19% CP (MR19; *n* = 30).

Fecal sodium levels did not differ between calves fed protein-reduced and conventional MR (MR22: 12.9 ± 15.9 g/kg DM; MR19: 11.2 ± 11.6 g/kg DM; *p* = 0.09). There was a moderate negative correlation between DM content and sodium content of the fecal samples in both groups (MR22: R = −0.516; *p* < 0.01 and MR19: R = −0.428; *p* < 0.01; Figure 3).

The sodium content was particularly high in the fecal samples with lower DM content but differed barely between medium and high DM content in the feces (Table 6).

### 3.5. Blood Parameters

#### 3.5.1. GH and IGF-1

The serum concentrations of growth hormone (GH) and insulin-like growth factor 1 (IGF-1) are shown in Table 7.

**Figure 4:**
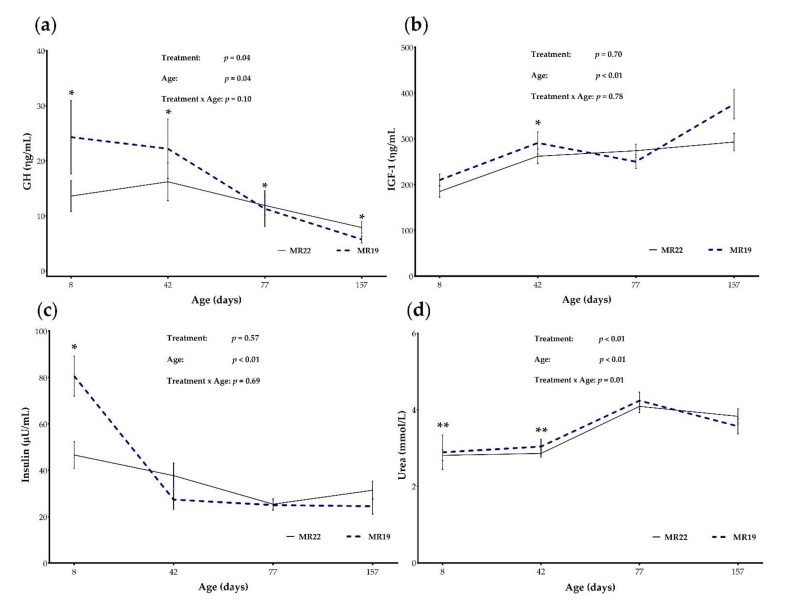
Blood metabolites represented as means and standard error bars of (**a**) GH, (**b**) IGF-1, (**c**) Insulin, and (**d**) Urea concentration of calves fed with milk replacer content 22% CP (MR22; *n* = 35) or 19% CP (MR19; *n* = 33), according to time periods. * Denotes difference with *p* < 0.05 or ** *p* < 0.01.

Serum levels of GH were significantly different between groups MR22 and MR19 during the entire study period (*p* < 0.05). At the age of 8 d (*p* < 0.01) and 42 d (*p* = 0.02), the GH levels were higher in MR19, while at the age of 77 d (*p* = 0.01) and 157 d (*p* = 0.04), the GH levels were significantly higher in groups MR22, resulting in a treatment (*p* = 0.04) and age effect (*p* = 0.04) and their interaction (*p* = 0.10; Figure 4a). In terms of IGF-1 concentrations, a significant difference between both treatments were only found at d 42 of life (MR22: 262 ± 93.6; MR19: 291 ± 136; *p* = 0.03). However, no effect of a treatment by age interaction was observed on IGF-1 concentration (*p* = 0.78; Figure 4b).

#### 3.5.2. Insulin and Glucose

To evaluate the metabolic situation, insulin, glucose, and urea concentration were determined in the serum. While at d 8 (start of experiment), the insulin levels were nearly twice as high in MR19 than in MR22 (80.6 vs. 46.6, respectively; *p* = 0.04, Table 7), the insulin levels at d 42, 77, and 157 barely differed (Figure 4c). However, there was no significant difference in glucose levels during the entire study period (*p* > 0.05; Table 7). Regarding the urea concentration, levels were higher during the pre-transition phase (from d 8 to d 42) in calves fed with MR19 (*p* < 0.001; Figure 4d). Moreover, a treatment × time interaction was found for the urea level during the entire experimental period (*p* = 0.01).

## 4. Discussion

Recent research on dairy calves has investigated management and nutrition improvements to benefit future productivity. Several studies have reported on the appropriate protein percentages in calf MR for optimal growth of young calves [34,35,36,37,38]. The high digestibility of protein content used for MR is a key factor in determining the outcome of the feeding program [4,26].

### 4.1. Performance of Calves

In the current study, MR intake as well as total DM intake were not dependent on protein concentration during the entire experimental period. Since a large proportion of the calves did not consume the entire 1600 g/d, it can be inferred that this amount was close to an ad libitum supply. Moreover, in the present study, during the transition phase (d 43-77) in both groups, the intake of MR was reduced. During the first six wk of the trial, MR19 calves showed greater intake of MCF than MR22 calves (146 vs. 91 g/d), even if this amount is about 7-11% of the MR intake and thus comparably low. However, Cowles et al. [39] also described a higher intake of starter when the protein content in the MR was reduced from 28% to 20%. Nonetheless, CP requirement of dairy calves for 50-kg Holstein calf amounts up to 262 g with ADG of 800 g according to NASEM [40], while the Society of Nutrition Physiology recommends 210 g CP for 50 kg dairy calves with a daily weight gain of 600 g and 345 g CP for 75 kg dairy calves with a daily weight gain of 800 g [41]. The CP intake via MR from d16-22 amounted on average 265 g/d (MR19) and 312 g/d (MR22; Table S4), respectively. During the transition phase (d43-77), mean BW was 94 kg and mean ADG was 1067 g/d. According to NRC [42] this results in a CP requirement of about 350 g/d for veal calves fed only MR and about 430 g/d for weaned (ruminant) calves. In the present study, mean total CP intake by MR and MCF during d43-77 amounted to 349 g/d (MR22) and 355 g/d (MR19), respectively. Considering the high daily gains, the protein requirement in this phase nevertheless seems to be covered despite the high solid feed intake. However, one limitation of the study is that intake of solid feed (MCF and TMR), in contrast to intake of MR, was only recorded on group basis. As animals differed in age, it is difficult to determine the total DM, CP and energy intake for single weeks. Therefore, total protein and energy supply were further assessed by means of metabolic traits (see Section 4.3).

The different level of CP in the MR did not affect weekly BW during the entire study. Consistent with this, the ADG did not differ between MR22 and MR19 during the entire trial and amounted to 860 g/d for MR22 and 831 g/d for MR19 between d1 and d77, which is in accordance with the NRC [42] model. Similarly, in the study by Jaeger et al. [36], feeding 24% CP in MR at a feeding rate of 680 g DM/d did not affect pre-transition BW gain (1 to 42 d) but improved postweaning ADG of dairy calves. Bartlett et al. [4] demonstrated that increasing dietary CP in MR increased lean tissue gain and might increase BW gain in calves fed 1.25% and 1.75% MR of BW (DM basis). In previous publications, it was reported that the ADG of the dairy calves between birth and two months of age was positively related to starter intake [26,43]. This is not in agreement with our results, as calves fed MR19 during the first 6 wks the intake of MCF was greater compared to group MR22; however, the total DM intake did not differ and no difference on ADG was observed. However, a substantial decrease in ADG was observed during 42–49 d. It could be hypothesized that calves were unable to compensate for the reduced nutrient from milk by increasing their intake of starter; therefore part of the growth advantage achieved before weaning could have been lost due to reduced consumption of nutrients [44,45]. Thus, the reduction in CP content of MR should be further evaluated in field studies on larger dairy farms. In calf rearing, on the one hand, satisfying the suckling need in terms of animal welfare and, on the other hand, increasing the intake of solid feed to promote the development of the forestomach is desired. In the present study, both objectives were achieved in a resource-efficient manner (CP reduction). Furthermore, performance of calves during the postweaning period suggests that protein intake did not limit growth in calves fed MR19 and our ADG results for the experimental period on days 43–77 suggest that the mean total CP intake in this period which was 349 g/d for MR22 and 355 g/d for MR19, respectively, adequately supplied the protein requirements of the animals. Regarding the growth development parameter in the current study, the heights at sacrum were not dependent on MR protein contents throughout the trial. Body measurements such as sacrum height are relative to skeletal size for any particular age of animal, but this does not necessarily imply a high association with muscling [46]. In a previous report, average height at sacrum was 126 cm at d 168 [46], while in our study, mean SH was 118 cm at d 157. Thus, there was no negative effect on growth performance and development in the calf when CP was reduced to 19% in MR.

### 4.2. Fecal Quality

The characteristics of feces might indicate the health status in young calves, and fecal consistency scoring is commonly used as a measure for diarrhea in most studies evaluating health in dairy calves [25,47,48]. In the present study, during the pre-transition and transition phase, MR22 calves significantly more often showed a feces of medium pulpy consistency (63.1% vs. 57.6%), which corresponds to the physiological condition according to Dirksen [49]. However, the groups did not differ significantly but only numerically regarding the frequency of loose and very loose feces. In contrast, a previous study reported that pre-weaned calves fed high CP (up to 438 g/d) had looser feces than calves fed CP 361 g/d [50]. However, Chapman et al. [26] found no differences in fecal scores when the calves were fed MR with CP intake 0.184 kg/d up to 0.245 kg/d. The cause of changes in fecal characteristics in this study were not determined, but preweaning calves are particularly susceptible to both infectious diarrhea and dietary scouring [51]. One reason for dietary souring is a putrefactive diarrhea due to high amounts or low prececal digestibility of CP [52]. However, CP source was the same for both MR19 and MR22. Another reason for dietary scouring is a fermentative diarrhea, which is caused by high amounts of carbohydrates. As the NfE fraction was 51.5 g/kg in MR22 and 56.0 g/kg in MR19, this should be considered as a possible influencing factor in the present study. Nevertheless, it cannot be conclusively determined why the fecal consistency of MR19 during the pre-transition and transition phase corresponded less often to the physiological state. In general, diarrhea occurred rarely in both groups and only two animals from each group required rehydration by electrolyte solution. The color of the feces is mainly influenced by the ingested feed. A brownish-green color shows that calves have been eating herbages as the chlorophyll from the plants causes the coloring of the feces [49]. While during the pre-transition and transition phase, in both groups fecal samples of a brown-orange and yellow-brown color occurred, in the postweaning phase (nearly) all fecal samples appeared in brownish-green color which indicates that calves successfully physiological changed in their digestive system.

Besides fecal consistency scoring, the moisture content of collected feces was determined. A DM content of feces less than 150 g/kg is considered as a criterion for diarrhea [49,53]. Overall fecal quality in terms of fecal DM during the entire experimental period was higher in calves fed MR22. This finding is not in agreement with a previous report that showed that protein fed in excess of requirements can result in more liquid feces [54]. Furthermore, fecal pH was found to be an indicator of pH status in the small intestine [55]. The pH can vary from greater than 7 to less than 5 depending on the type of diet fed to ruminants [56]. The consequence of microbial feed fermentation is a production of acids such as lactate, acetate, propionate, and butyrate [44]. In the present study, a significant effect of MR on fecal pH was observed. The group fed high protein content (MR 22) showed a higher fecal pH profile than calves fed MR19. This is in agreement with Haaland et al. [57] and Veira et al. [58], who found that fecal pH values rose when increasing the protein content in the diet. Apart from the above mentioned reason, the increase in fecal pH might be due to calcium in the diets [55]. Our findings showed a slightly higher calcium content in MR22 than MR19 (8.70 vs. 7.90 g/kg DM). Moreover, Wheeler and Noller [59] found that a low fecal pH was determined when ruminants were offered high concentrate rations, which is equivalent to high NfE amounts. This is in agreement with our findings, since a lower pH profile was observed in MR19 calves at age 42d (7.49 vs. 8.00) that showed on the one hand a greater intake of MCF during the first 42d of age (146 vs. 91 g/animal/d) and on the other hand a higher intake of NfE due to MR. Both factors (higher intake of MCF and higher amount of NfE in MR19) could have reduced fecal pH. Regarding the sodium content of fecal samples, a moderate correlation between DM content and sodium content was observed in both groups (MR22: R^2^ = 0.516 and MR19: R^2^ = 0.428). The sodium content was particularly high in the fecal samples with lower DM content. Nevertheless, it should be noted that correlation analyses are particularly suitable for large sample sizes which may limit the power of our findings [60]. In the case of diarrhea, i.e., the worst fecal consistency score, high electrolyte levels are excreted in feces [61]. The calf must therefore be constantly observed to check whether the fluid and electrolyte levels can be balanced through the MR. However, the CP-reduced MR with feeding high amounts up to 10 L did not influence the fecal quality or general health of the calves in this study. It goes without saying that the health of calves does not only depend on nutrition, but also on other environmental factors such as housing.

### 4.3. Metabolic Traits

The GH and IGF-1 levels were used to assess growth potential. The GH-IGF-1 axis or the somatotropic axis is already functional in the young calf and can be used for assessment [62,63]. The somatotropic axis controls growth and is influenced by the interactions between GH and IGF-1 [64]. The pattern of GH secretion is markedly affected by dietary treatment. Bassett et al. [65] described that plasma GH concentrations were negatively correlated with the amount of CP in ruminants’ diet. Likewise, our own recent research provided evidence that greater GH levels were observed in group MR19 that pre-transition (d8-42) consumed less CP than group MR22 (276 vs. 311 g/d). In contrast, Hammon et al. [66] found that the GH concentrations were not influenced the feeding level of MR, i.e., ad libitum or restrictive supply, so that the simultaneous increase in protein and energy intake showed no effect on GH concentrations.

On the basis of the results that a high concentration of IGF-1 increased over the whole growth period in both groups, it can be assumed that calves which ingested smaller amounts of CP had no disadvantage in terms of nutrient supply or immune status. Previous studies [67,68] showed that increasing CP intake stimulated increased concentrations of IGF-1 in the plasma of calves, while a study with different weaning regimes proved that IGF-I-concentrations cannot be attributed to differences in protein supply [69]. However, a difference in terms of IGF-1 concentrations was found at d 42 of age (MR19: 291 vs. 262 ng/mL in MR22).

Serum urea levels were also recorded to assess protein supply. During the postweaning phase, no significant differences were found dependent on protein concentration. However, during the pre-transition phase, the urea concentration was higher for calves fed with MR19. The lower serum urea concentration in the 22% CP calves indicates that they might have had a greater body protein accretion and the proportion of amino acids used for energy supply was lower than in the 19% CP calves. Nevertheless, on average, the values varied between 2.8 mmol/L and 4.2 mmol/L, which is within the reference range from 2.6 to 6.6 mmol/L according to Fürll [70]. While an excess of blood urea can result from a nutritional energy deficit or cardiovascular shock, low blood urea levels can be caused by protein deficiency or chronic diseases [70]. Urea levels show that neither group was deficient in energy or protein. This indicates that the use of a MR reduced in CP content is both practicable and sustainable. For future studies, it would be interesting to determine if a reduction in protein concentration of MR is associated with less muscle growth in favor of body fat percentage in calves. Differences in body composition could explain that no difference in body weight was detected despite the higher protein intake (and lower urea level at the same time). However, as this study did not investigate body compensation, this cannot be substantiated.

As calves grow older and solid feed intake increases, ruminal fermentation is established, and decreased blood glucose and insulin concentrations are observed [71]. This is due to hepatic gluconeogenesis [44]. In the literature, it is stated that the insulin content in the serum of calves should drop at the age of 45 d and then rise again by the 90th d of life [71]. Regardless of the mechanisms involved in insulin secretion in dairy calves, previous reports stated that higher insulin levels may favor growth performance of growing calves [72]. In calves, the glucose content in the blood decreases with increasing age [71,73]. Accordingly, mean blood glucose declined from 119 mg/dL at 8 d of age to 84.3 mg/dL in the five-month-old calves. However, in agreement with Makizadeh et al. [72], in this study the dietary protein content had no effect on the blood glucose level. It should nevertheless be noted that glucose levels in the blood fluctuate during the day and are strongly dependent on the time interval between the last feed intake [74,75]. Although blood sampling was performed always to the same time, the calves had access to feed at all times. Therefore, blood sampling cannot be considered in exact relation to feed intake. Yet, this source of error is the same for both groups. Overall, and based on the data in our study, reducing protein content in the milk replacer had no negative effect for the calves due to metabolic traits.

## 5. Conclusions

The results of our study suggest that a milk replacer with a 19% CP content, offered in liberal amounts during the first 6 weeks of life, was sufficient for calves. Calves consumed about 8.1L of a 16% DM milk; a CP intake of 250–270 g until day 42 and 350–360 g at 8–10 weeks of age is obviously adequate.

## Figures and Tables

**Table 1:** List of ingredients, energy content and chemical composition as analyzed (% of DM unless stated) of milk replacers (MR) containing two different levels of protein.

Item	MR22 ^1^	MR19 ^2^
**Ingredient**		
Skimmed milk powder	40.0	40.0
Whey powder	12.5	32.5
Whey powder, partially delactosed	10.0	-
Whey protein powder	10.0	-
Vegetable fat (coconut/palm)	18.0	18.0
Beer yeast	0.30	0.30
Pregelatinized wheat starch	2.00	2.00
Dextrose	1.50	1.50
**Chemical composition**		
DM (% of fresh matter)	96.3	96.8
Crude ash	7.20	6.70
Crude protein	21.8	18.5
Ether extract	19.5	18.8
Crude fibre	0.00	0.00
NfE ^3^	51.5	56.0
Metabolizable energy (ME), MJ/kg of DM	17.5	17.4
Protein:energy ratio MJ ME/g CP	1.25	1.06
Calcium	0.87	0.79
Phosphorus	0.71	0.65
Sodium	0.68	0.58
Potassium	1.57	1.31
Magnesium	0.17	0.14

^1^ MR designed to contain 22% crude protein; ^2^ MR designed to contain 19% crude protein; ^3^ NfE, nitrogen-free extract.

**Table 2:** Energy content and chemical composition (% DM unless stated) of concentrates, straw, mixed calf feed (MCF; 87% concentrates and 13% straw), and total mixed ration (TMR) was analyzed.

Item	Concentrates	Straw	MCF	TMR
DM (% of fresh matter)	87.5	89.6	87.8	46.2
Metabolizable energy (ME), MJ/kg of DM	13.0	6.40	12.2	10.9
Crude ash	7.40	4.70	7.00	8.90
Crude protein	21.1	3.00	18.7	15.3
Ether extract	4.90	1.20	4.40	3.50
Crude fibre	6.30	46.5	11.5	18.9
NfE ^1^	60.4	44.7	58.4	53.4

^1^ NfE, nitrogen-free extract.

**Table 3:** Dry matter (DM) intake (g/d) differentiated according to experimental periods and feed sources given in g/animal/d (mean ± SD).

Age (d)	DM intake	MR22 ^1^	MR19 ^2^	*p*-Value
8–42	MR	1310 ± 137	1300 ± 137	0.48
	MCF	91.0 ± 66.0	146 ± 120	<0.01
	Total	1401 ± 176	1446 ± 218	0.73
43–77	MR	555 ± 345	560 ± 353	0.57
	MCF	1217 ± 664	1344 ± 677	0.43
	Total	1772 ± 339	1904 ± 317	0.06
78–157	TMR	2627 ± 574	2547 ± 564	0.81
	MCF	1470 ± 140	1532 ± 154	0.35
	Total	4097 ± 893	4079 ± 730	0.85

MR: milk replacer. MCF: mixed calf feed. TMR: total mixed ration; ^1^ MR designed to contain 22% crude protein; ^2^ MR designed to contain 19% crude protein.

**Table 4:** Sacral Height (mean ± SEM; min-max) of groups MR22 and MR19 depending on age (d), given in cm.

Age (d)	MR22 ^1^		MR19 ^2^		*p*-Value
	Mean ± SEM	Min-Max	Mean ± SEM	Min-Max	
Initial	82.3 ± 056	76–88	81.9 ± 0.57	74–88	0.46
8	84.4 ± 0.50	78–89	84.3 ± 0.54	79–92	0.75
22	88.5 ± 0.43	82–93	88.8 ± 0.49	82–94	0.56
42	94.3 ± 0.48	87–100	94.2 ± 0.46	87–99	0.74
77	102 ± 0.43	95–107	103 ± 0.50	96–108	0.50
117	111 ± 0.49	104–117	110 ± 0.61	105–119	0.32
157	118 ± 0.60	109–123	118 ± 0.64	112–124	0.68

^1^ MR designed to contain 22% crude protein; ^2^ MR designed to contain 19% crude protein.

**Table 5:** Characteristics of fecal samplings during the pre-transition and transition phase as well as the postweaning phase depending on crude protein content of milk replacer (MR) given in percent and number of observations (*n*).

Characteristic	Pre-Transition ^3^ and Transition ^4^ PHASE		*p*-Value	Postweaning ^5^ Phase		*p*-Value
	MR22 ^1^ (*n* = 350)	MR19 ^2^ (*n* = 330)		MR22 ^1^ (*n* = 102)	MR19 ^2^ (*n* = 99)	
**Color**						
Brown-green	60.6% (212)	54.9% (181)	0.20	99.0% (101)	100% (99)	0.32
Brown-orange	16.9% (59)	16.1% (53)	0.79	1.00% (1)	0% (0)	0.46
Yellow-brown	22.5% (79)	28.8% (95)	0.26	0% (0)	0% (0)	-
Whitish-grey	0% (0)	0.20% (1)	0.81	0% (0)	0% (0)	-
**Consistency**						
Firm	0% (0)	0% (0)	-	0% (0)	0% (0)	-
Thick Pulpy	2.00% (7)	1.82% (6)	0.67	17.7% (18)	5.05% (5)	0.02
Medium Pulpy (Pasty)	63.1% (221)	57.6% (190)	0.02	68.6% (70)	75.8% (75)	0.74
Mushy	20.0% (70)	21.5% (71)	0.76	11.8% (12)	16.2% (16)	0.58
Loose	9.70% (34)	9.70% (32)	0.64	1.96% (2)	1.01% (1)	0.70
Watery	5.14% (18)	9.39% (31)	0.25	0% (0)	2.02% (2)	0.71

^1^ MR designed to contain 22% crude protein; ^2^ MR designed to contain 19% crude protein; ^3^ Pre-transition phase from d8 to d42; ^4^ Transition phase from d43 to d77; ^5^ Postweaning phase from d78 to d157.

**Table 6:** Dry matter (DM) content (g/kg) and sodium content (g/kg DM) depending on fecal DM classification and crude protein content of MR (mean ± SD).

Fecal DM Classification	MR22 ^1^		MR19 ^2^	
	DM Content	Sodium Content	DM Content	Sodium Content
Low	44.7 ± 11.4	31.4	51.4 ± 7.48	22.9
Medium	165 ± 0.57	4.27	153 ± 1.10	5.82
High	284 ± 29.4	3.95	236 ± 12.7	4.85

^1^ MR designed to contain 22% crude protein; ^2^ MR designed to contain 19% crude protein.

**Table 7:** Serum levels of growth hormone (GH), insulin-like growth factor 1 (IGF-1), insulin, glucose, and urea depending on age and crude protein content of MR (mean ± SEM).

Item	Treatment				*p*-Value
	MR22 ^1^	SEM	MR19 ^2^	SEM	Treatment
**GH, ng/mL**					
d 8	13.6 ^b^	2.84	24.3 ^a^	6.67	<0.01
d 42 ^3^	16.2 ^b^	3.44	22.2 ^a^	5.39	0.02
d 77 ^4^	11.9 ^a^	1.75	11.3 ^b^	3.25	0.01
d 157	7.89 ^a^	1.05	5.73 ^b^	0.63	0.04
**IGF-1, ng/mL**					
d 8	185	12.6	210	13.2	0.92
d 42 ^3^	262 ^b^	15.8	291 ^a^	24.1	0.03
d 77 ^4^	274	14.1	250	15.2	0.75
d 157	293	19.2	376	32.1	0.07
**Insulin, µU/mL**					
d 8	46.6 ^b^	5.76	80.6 ^a^	8.63	0.04
d 42 ^3^	37.7	5.42	27.4	4.27	0.11
d 77 ^4^	25.4	2.44	25.0	2.19	0.49
d 157	31.4	3.94	24.5	3.37	0.48
**Glucose, mg/dL**					
d 8	108	7.75	132	6.67	0.43
d 42 ^3^	109	4.90	88.4	4.24	0.50
d 77 ^4^	79.9	3.00	73.4	2.16	0.22
d 157	78.5	1.94	90.4	2.03	0.94
**Urea, mmol/L**					
d 8	2.81 ^b^	0.14	2.89 ^a^	0.45	<0.01
d 42 ^3^	2.86 ^b^	0.10	3.04 ^a^	0.19	<0.01
d 77 ^4^	4.09	0.16	4.24	0.22	0.12
d 157	3.83	0.20	3.57	0.20	0.83

^a,b^ Superscript letters indicate significant differences between groups MR22 and MR19; ^1^ MR designed to contain 22% crude protein; ^2^ MR designed to contain 19% crude protein; ^3^ Pre-transition phase from d8 to d42: 10L of MR at a concentration of 160 g/L; ^4^ Transition phase from d43 to d77: linear reduction from 10L to 0L at a concentration of 125 g/L.

## Data Availability

The data presented in this study are available in this manuscript and Supplementary Materials.

## Supplementary Materials

The following supporting information can be downloaded at: https://www.mdpi.com/article/10.3390/ani12141756/s1, Table S1: Declared chemical composition and feed additives of MR22 and MR19, Table S2: Colostrum and whole milk intake (mean ± SD) of groups MR22 and MR19 during the week of life, given in L/calf/d, Table S3: Sacral Height (mean ± SEM; min-max) of groups MR22 and MR19 depending on age (d), given in cm, Table S4: Weekly intake of dry matter (DM, mean ± SD) and crude protein (CP, mean ± SD) by milk replacer of groups MR22 and MR19 during pre-transition and transition period, given in g/calf/d.
