# Supplementary material for: Impacts of Reducing Protein Content in Milk Replacer on Growth Performance and Health of Young Calves
Source: Animals (Basel). 2022 Jul 8;12(14):1756. doi: 10.3390/ani12141756 (PMC9312336; doi:10.3390/ani12141756)
Supplement: Supplementary file 1 [file animals-12-01756-s001.zip › animals-1742746-supplementary.pdf]

Supplementary material

Table S1. Vitamin concentration of vitamin B complex (Vitamin-B-Komplex pro inj., Serumwerk Bernburg AG)

Vitamin	Concentration (mg / mL)
Thiamine chloride hydrochloride	8.0
Cyanocobalamine	0.02
Nicotinamide	20.0
Pyridoxine hydrochloride	0.8
Riboflavin phosphate sodium	0.42
Dexpanthenol	0.84

Table S2. Declared chemical composition and feed additives of MR22 and MR19.

Item	Unit	MR22	MR19
Crude ash	%	7.50	6.80
Crude Protein	%	22.0	19.0
Lysine	%	1.80	1.65
Ether extract	%	18.0	18.0
Crude fibre	%	0.10	0.10
Calcium	%	0.80	0.75
Phosphorus	%	0.75	0.65
Magnesium	%	0.20	0.20
Sodium	%	0.70	0.60
Vitamin A	I.E./kg	100,000	100,000
Vitamin D_3	I.E./kg	10,000	10,000
Vitamin E	mg/kg	200	200
Vitamin K	mg/kg	5.00	5.00
Vitamin C	mg/kg	450	450
Vitamin B_1	mg/kg	15.0	15.0
Vitamin B_2	mg/kg	24.0	24.0
Vitamin B_6	mg/kg	6.60	6.60
Vitamin B_{12}	mg/kg	66.0	66.0
Niacin	mg/kg	100	100
Pantothenic acid	mg/kg	50	50
Choline chloride	mg/kg	313	313
Iron	mg/kg	90.0	90.0
Copper	mg/kg	9.00	9.00
Zinc	mg/kg	37.0	37.0
Manganese	mg/kg	28.0	28.0
Iodine	mg/kg	0.40	0.40
Selenium	mg/kg	0.20	0.20
Pucoferm[1]	10^9 CFU	1.00	1.00

[1] Enterococcus faecium M74 (NCIMB 11181, 4b1708), Pulte GmbH & Co. KG, Prien, Germany

Table S3. Colostrum and whole milk intake (mean ± SD) of groups MR22 and MR19 during the first week of life, given in L/calf/d.

Day of life	MR22	MR19
Birth	3.9 ± 1.7	3.5 ± 2.0
1	4.4 ± 1.4	4.9 ± 1.7
2	5.5 ± 1.4	6.5 ± 1.4
3	7.1 ± 1.0	7.2 ± 1.3
4	8.3 ± 1.2	8.0 ± 1.3
5	8.5 ± 1.6	8.6 ± 1.1
6	7.9 ± 3.2	7.6 ± 2.4
7	4.8 ± 2.6	5.2 ± 2.9

Table S4. Weekly intake of dry matter (DM, mean ± SD) and crude protein (CP, mean ± SD) by milk replacer of groups MR22 and MR19 during pre-transition and transition period, given in g/calf/d.

Trial period	Age (days)	DM		CP	
		MR22	MR19	MR22	MR19
Pre-transition	9-15	1060 ± 209	1042 ± 239	231 ± 45.5	193 ± 44.2
	16-22	1388 ± 205	1383 ± 243	303 ± 44.8	256 ± 45.0
	23-29	1434 ± 184	1433 ± 165	313 ± 40.1	265 ± 30.5
	30-36	1426 ± 158	1408 ± 209	311 ± 34.4	261 ± 38.7
	37-42	1447 ± 148	1397 ± 196	315 ± 32.3	259 ± 36.3
Transition	43-49	1037 ± 46.7	1035 ± 60.7	226 ± 10.2	192 ± 11.2
	50-56	772 ± 24.8	775 ± 27.7	168 ± 5.40	143 ± 5.13
	57-63	470 ± 7.46	462 ± 19.5	102 ± 1.62	85.4 ± 3.61
	64-70	149 ± 18.6	140 ± 13.2	32.6 ± 4.05	25.9 ± 2.44
